# Functional and Expressional Analyses Reveal the Distinct Role of Complement Factor I in Regulating Complement System Activation during GCRV Infection in *Ctenopharyngodon idella*

**DOI:** 10.3390/ijms231911369

**Published:** 2022-09-26

**Authors:** Yi Liu, Zhao Lv, Tiaoyi Xiao, Xuewen Zhang, Chunhua Ding, Beibei Qin, Baohong Xu, Qiaolin Liu

**Affiliations:** Hunan Engineering Technology Research Center of Featured Aquatic Resources Utilization, Hunan Agricultural University, Changsha 410128, China

**Keywords:** grass carp, complement factor I, complement component 3b, grass carp reovirus infection, complement regulation mechanism

## Abstract

Complement factor I (CFI), a complement inhibitor, is well known for regulating the complement system activation by degrading complement component 3b (C3b) in animal serum, thus becoming involved in innate defense. Nevertheless, the functional mechanisms of CFI in the complement system and in host-pathogen interactions are far from being clarified in teleost fish. In the present study, we cloned and characterized the CFI gene, CiCFI, from grass carp (*Ctenopharyngodon idella*) and analyzed its function in degrading serum C3b and expression changes after grass carp reovirus (GCRV) infection. The open reading frame of CiCFI was found to be 2121 bp, encoding 706 amino acids with a molecular mass of 79.06 kDa. The pairwise alignments showed that CiCFI shared the highest identity (66.9%) with CFI from *Carassius gibelio* and the highest similarity (78.7%) with CFI from *Danio rerio*. The CiCFI protein was characterized by a conserved functional core Tryp_SPc domain with the catalytic triad and substrate binding sites. Phylogenetic analysis indicated that CiCFI and the homologs CFIs from other teleost fish formed a distinct evolutionary branch. Similar with the CFIs reported in mammals, the recombinant CiCFI protein could significantly reduce the C3b content in the serum, demonstrating the conserved function of CiCFI in the complement system in the grass carp. CiCFI mRNA and protein showed the highest expression level in the liver. After GCRV infection, the mRNA expressions of CiCFI were first down-regulated, then up-regulated, and then down-regulated to the initial level, while the protein expression levels maintained an overall downward trend to the late stage of infection in the liver of grass carps. Unexpectedly, the protein levels of CiCFI were also continuously down-regulated in the serum of grass carps during GCRV infection, while the content of serum C3b proteins first increases and then returns to the initial level, suggesting a distinct role of CiCFI in regulating complement activation and fish-virus interaction. Combining our previous results that complement factor D, a complement enhancer, shows continuously up-regulated expression levels in grass carps during GCRV infection, and this study may provide the further essential data for the full picture of complex complement regulation mechanism mediated by Df and CFI of the grass carp during pathogen infection.

## 1. Introduction

As the humoral backbone, the complement system serves as the first line of innate immune defense against invading pathogens in all vertebrates [1,2]. This system consists of more than 35 plasma proteins or membrane-bound proteins that can precisely surveil and remove the invading pathogens and altered host cells after activation [1,2,3,4]. According to the category of initial stimulus, the complement system can be activated through three pathways: the classical pathway (CP), the lectin pathway (LP), and the alternative pathway (AP) [1,5]. The cleavage of complement component 3 (C3) is the central step for all the three activation pathways [6,7], where C3b, the fragment of C3, is generated for the subsequent serial cascade activation involving C5–C9 and the formation of membrane attack complex (MAC) [1,8]. With the numerous components mediating three activation pathways, the complement system exerts high efficiency in eliminating foreign pathogens, while its inappropriate and excessive activation can provoke autoimmune diseases in hosts [9]. Correspondingly, animal hosts are equipped with various complement inhibitors that avoid the amplification of complement cascades [10,11].

The complement factor I (CFI), a serine protease, is one of the important complement inhibitors well-known for its role in the regulation of complement activation [12,13,14]. Structurally, the CFI protein has a molecular weight of about 88 kDa and consists of a heavy chain and a light chain connected via a single disulfide bond [15]. The heavy chain includes an N-terminal region, a factor I membrane attack complex (FIMAC) domain, a scavenger receptor cysteine-rich (SR) domain, two class A low-density lipoprotein receptor domains (LDLa1 and LDLa2), and a C-terminal region with unknown function; the light chain is composed of a trypsin-like serine protease (Tryp_SPc) domain [16]. The heavy chain determines the substrate recognition specificity for CFI, while the light chain plays an essential role in proteolytic activity [15]. In fact, CFI is mainly synthesized in the liver of animals and can also be detected in diverse cells including fibroblasts, keratinocytes, and mononuclear cells, while it generally exerts biological functions in animal plasma [17]. CFI precisely regulates the host’s complement system-mediated immune responses by cleaving and inactivating the plasma C3b that is involved in the assembly of the C3 and C5 convertases [9,12,15]. Mechanistically, the CFI-mediated C3b cleavage is a stepwise process and depends on the substrate modulating cofactors, such as complement factor H, C4bp, and CR1. In the presence of cofactors and CFI, C3b is first cleaved into iC3b and C3f, and then iC3b is cleaved into C3dg and C3c [12,13].

In mammals, numerous studies have already shown that CFI plays a crucial role in host-pathogen interactions. In humans, for example, CFI-mediated cleavage of HIV-hijacked C3b would block the interaction of HIV with the C3b receptor and CR1 protein on the surface of CD4^+^ T cells to reduce viral entry, which attenuates HIV infection [18]. Most patients suffering from CFI deficiency are susceptible to pulmonary, meningeal, or septicemic inflammatory diseases caused primarily by *Streptococcus pneumoniae*, *Neisseria meningitidis*, and *Haemophilus influenzae* infection [19]. Meanwhile, CFI has also been reported to assist the pathogen invasions. CFI can cleave C3b proteins bound onto the *Staphylococcus aureus* surface to decrease host phagocytosis efficiency, resulting in bacterial evasion from complement-mediated killing [20]. In critical COVID-19 patients, CFI is hijacked by SARS-CoV-2, which causes the overactivation of the complement system, excessive inflammatory response, and vascular dysfunction [21]. The evidence demonstrates that CFI can be a double-edged sword for mammals during pathogen infection, as it is involved in both host immune defense and pathogen evasions.

In teleost fish, CFI genes from *Ictalurus punctatus* [22], *Pelteobagrus vachellii* [23], *Cynoglossus semilaevis* [24], *Oncorhynchus mykiss* [25], *Paralichthys olivaceus* [26] and *Cyprinus carpio* [27] have been identified, and structural information and the mRNA expression profiles during pathogen infection are revealed in these studies. In *P. vachellii*, the mRNA expression levels of CFI in the liver, spleen and head kidney are first up-regulated and then down-regulated by *Aeromonas hydrophila* challenge [23]. CFI mRNA expressions are significantly increased in the liver of *C*. *semilaevis* after *Vibrio anguillarum* challenge [24]. During *Edwardsiella tarda* infection in *P. olivaceus*, CFI mRNA expressions peak significantly at 6 h post-infection in the spleen and kidney, decreasing at later infection hours [26]. These mRNA expression changes strongly suggest the involvement of CFI in fish-pathogen interactions. Scattered studies have also uncovered that the recombinant CFI proteins exhibit an antibacterial effect in *C*. *semilaevis* and *P. olivaceus* [24,26]. Nevertheless, the functional mechanisms of CFI in the complement system and in host-pathogen interactions are far from being clarified in teleost fish.

Grass carp, *Ctenopharyngodon idella*, is an important cultured freshwater fish in China, and its production reached 5.57 million tons in 2020 [28]. Nevertheless, the healthy aquaculture of grass carp is restricted long-term by grass carp hemorrhage disease (GCHD) caused by grass carp reovirus (GCRV) infection [29,30,31,32]. Previous studies have revealed that the AP is significantly activated in grass carp after GCRV infection with transcriptome analysis, with a 2.18-fold up-regulation of complement factor D (Df) mRNA expression and a 9410.14-fold down-regulation of CFI mRNA expression [33]. Df is a serine protease for activating the AP by cleaving complement factor B (Bf) to promote the cleavage of C3 [34], and its enhancement on complement activation has recently been verified in *C. idella* [35]. The aims of the present study are (1) to clarify the molecular characteristics of CFI (designated as CiCFI) from *C. idella*, (2) to verify the function of CiCFI in the complement system, and (3) to explore its role during GCRV infection, which will add fundamental data for the full picture of the complement regulatory mechanism mediated by Df and CFI during GCRV infection.

## 2. Results

### 2.1. Sequence Feature of CiCFI

The full-length cDNA sequence of CiCFI (GenBank accession number: ADN22949.1) was 2359 bp, and consisted of a 2121 bp open reading frame (ORF), a 107 bp 5′-terminal untranslated region (UTR), and a 131 bp 3′-UTR. The CiCFI ORF encoded a polypeptide of 706 amino acids, with a predicted molecular weight of 79.06 kDa and a theoretical isoelectric point of 6.34.

**Figure 1:**
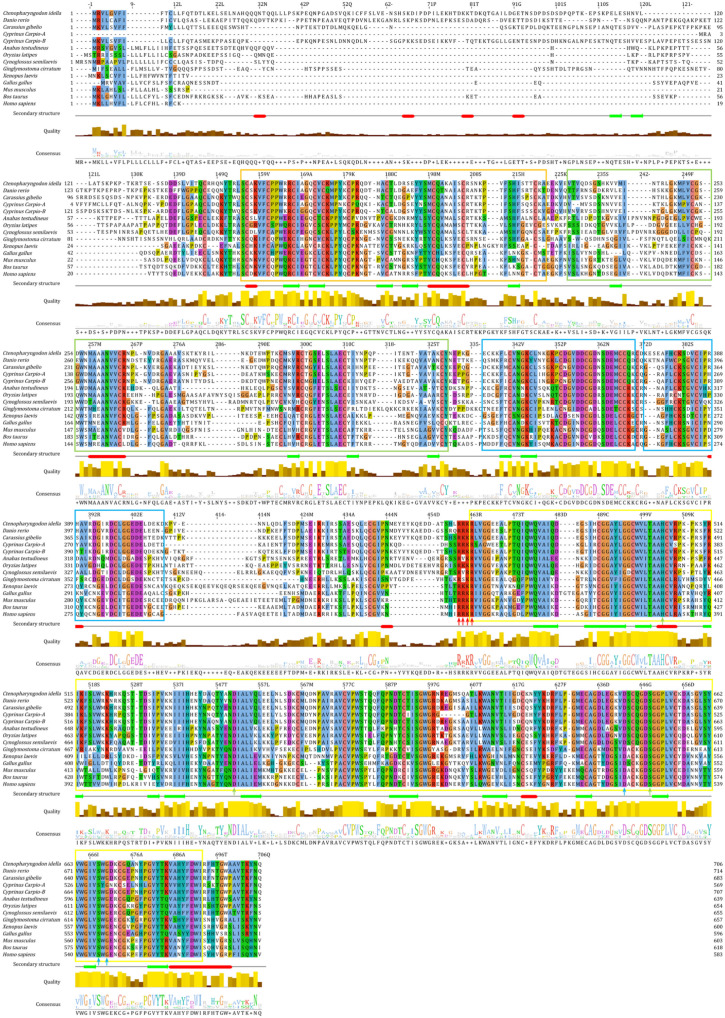
Multiple alignments of the deduced amino acid sequences of CiCFI with other species. The FIMAC, SR, LDLa1, LDLa2 and Tryp_SPc domains are marked by an orange box, green box, blue box, and yellow box, respectively. The cleavage site between a heavy chain and a light chain, the active site forming a catalytic triad, and the conserved substrate binding site are marked by red arrows, green arrows and blue arrows, respectively. Secondary structural features of CiCFI are presented below each amino acid sequence: red solid box, α-helix; green solid arrow, β-strand.

Multiple amino acid sequence alignments showed that CFI amino acid sequences from *C. idella* and thirteen other species were highly conserved, especially in 37 cysteine residues (Figure 1). In addition, CiCFI had the typical cleavage site of ^460^Arg–^461^Arg–^462^Lys–^463^Arg (RRKR) (Figure 1), which was reported to link the heavy chain and light chain, similar to other CFIs. Moreover, three residues of the catalytic triad (^504^His, ^551^Asp, and ^648^Ser) and three residues of the substrate binding site (^642^Asp, ^668^Ser, and ^670^Gly) were highly conserved among the fish CFI counterparts (Figure 1).

**Figure 2:**
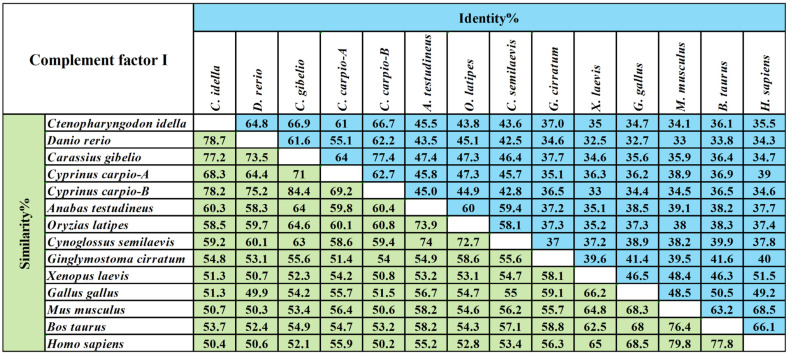
The amino acid sequence identity and similarity for the amino acid sequences of CFI from different species. The values of similarity and identity are backgrounded in green and blue, respectively.

A pairwise alignment of the CiCFI protein sequence with 13 other CFIs revealed that the deduced amino acid sequence of CiCFI shared 34.1–66.9% identity and 50.4–78.7% similarity with those of the known CFIs (Figure 2). Of these, CiCFI shared the highest identity (66.9%) with the *Carassius gibelio* CFI homolog and the highest similarity (78.7%) with the *Danio* *rerio* CFI homolog (Figure 2). 

### 2.2. The Predicted Domain Architecture and Three-Dimensional Structure Characteristic of CiCFI

**Figure 3:**
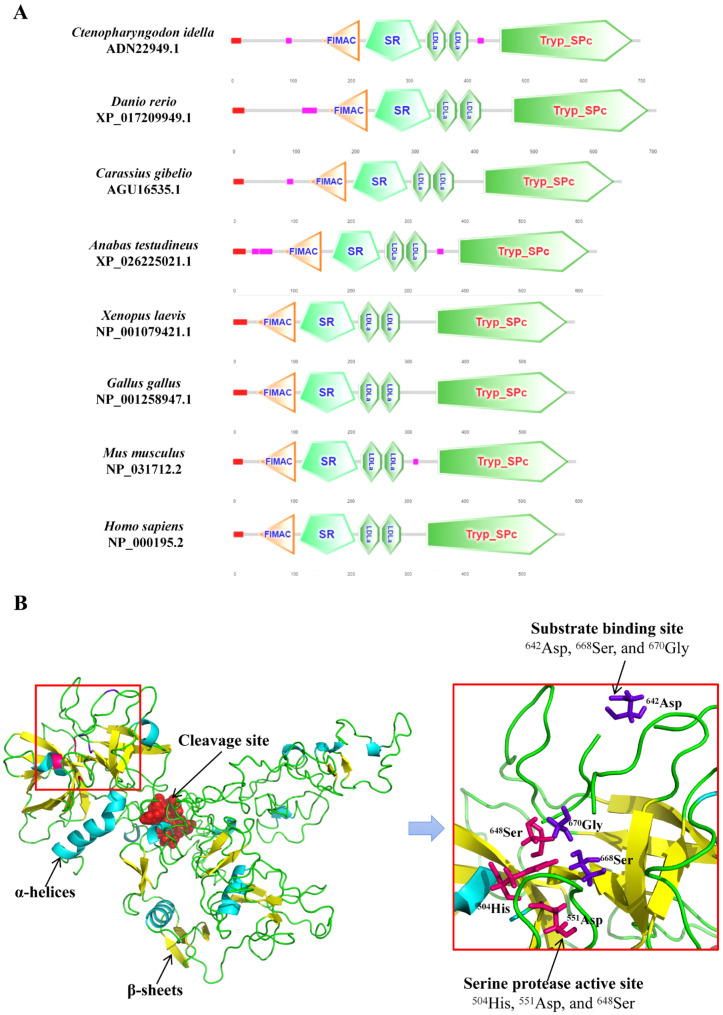
The predicted domain architecture and three-dimensional structure of CiCFI. (**A**) The predicted domain architectures of CFIs from *C. idella*, *D. rerio*, *C. gibelio*, *A. testudineus*, *X. laevis*, *G. gallus*, *M. musculus*, and *H. sapiens* were compared. FIMAC, SR, LDLa, and Tryp_SPc represent the abbreviations for factor I membrane attack complex, Scavenger receptor Cys-rich, low-density lipoprotein receptor domain class A, and trypsin-like serine protease, respectively. The scale on the bottom indicates the amino acid number. (**B**) In the predicted three-dimensional structure of CiCFI, the cleavage site is marked in red spheres, serine protease active site is colored in magenta, and substrate binding site is colored in purple.

The predicted domain architecture comparison analysis showed that the CiCFI contained conserved domains including FIMAC domain, SR domain, LDLa1 domain, LDLa2 domain and Tryp_SPc domain, which was similar to these CFIs from *D. rerio*, *C. gibelio*, *Anabas testudineus*, *Xenopus laevis*, *Gallus gallus*, *Mus musculus* and *Homo sapiens* (Figure 3A). The predicted three-dimensional structure analysis displayed that the CiCFI protein was composed of 12 α-helices and 26 β-sheets (Figure 3B). The locations of the conserved catalytic triad (^504^His, ^551^Asp, and ^648^Ser) and the conserved substrate binding site (^642^Asp, ^668^Ser, and ^670^Gly) of CiCFI were very close (Figure 3B).

### 2.3. The Phylogenetic Tree of CFIs

**Figure 4:**
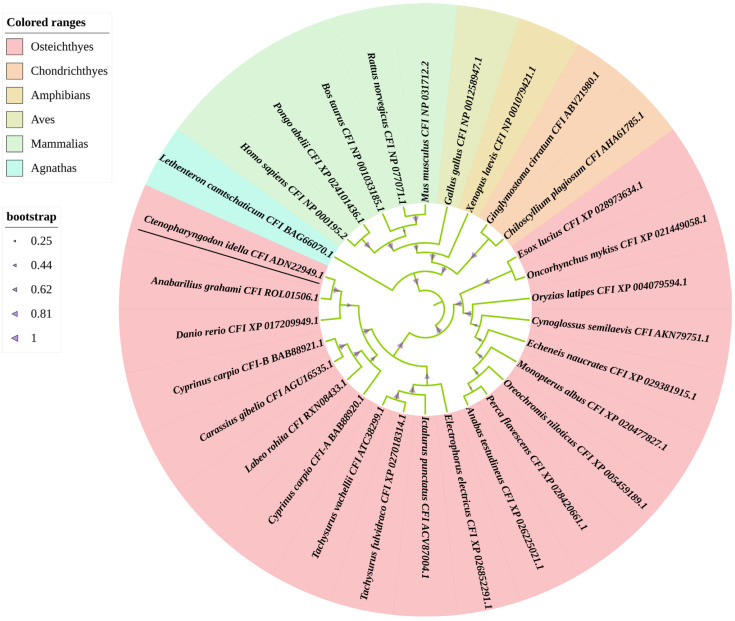
Phylogenetic tree constructed by NJ method for CFIs from various vertebrates. The CiCFI protein is underlined. All of the selected CFI proteins are separated into six branches that are marked by different colors.

To determine the evolutionary feature of the CiCFI protein, a phylogenetic tree was constructed based on full-length CFI amino acid sequences from various vertebrates. The phylogenetic tree showed that the selected 30 CFI homologs could be divided into six groups, containing Osteichthyes, Chondrichthyes, Amphibians, Aves, Mammalias and Agnathas branches (Figure 4). The CiCFI was first clustered with those CFIs from Cypriniformes including *Anabarilius grahami*, *D. rerio*, *C. carpio*, *C. gibelio* and *Labeo rohita*, and then clustered with the CFIs mainly from Siluriformes, Salmoniformes, and Perciformes, into the branch of Osteichthyes (Figure 4).

### 2.4. The Activity of CiCFI to Degrade CiC3b

**Figure 5:**
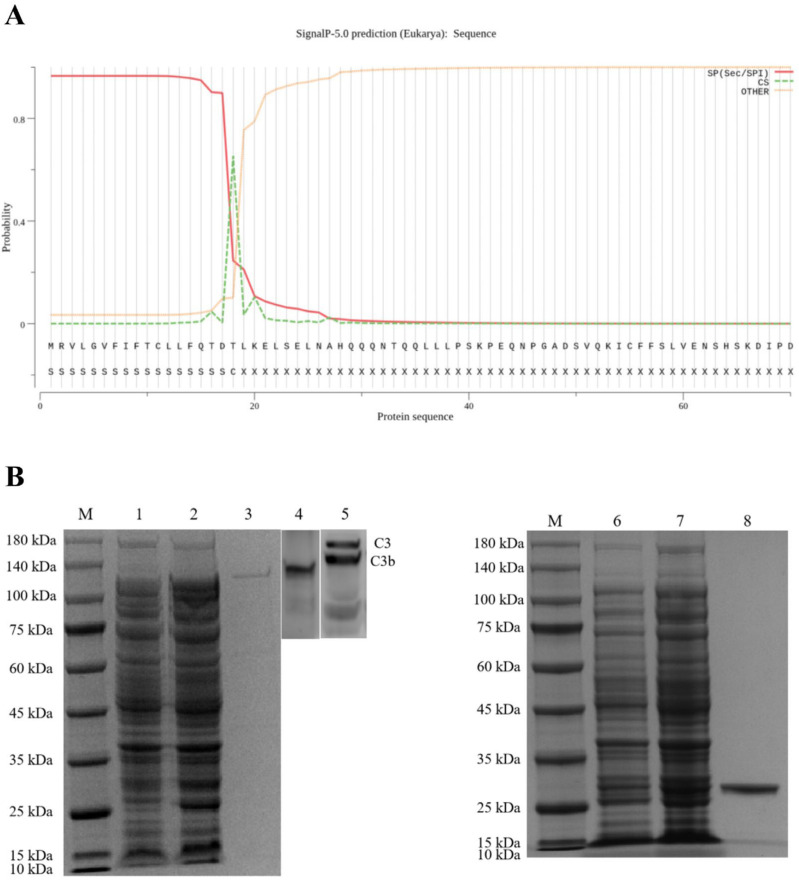
The rCiCFI protein and verification of rabbit anti-CiCFI and -CiC3 polyclonal antibodies. (**A**) The first 18 amino acid residues were predicted to form a signal peptide in the CiCFI protein by using the SignalP-5.0 tool. (**B**) Detection of rCiCFI protein and verification of rabbit anti-CiCFI and -CiC3 polyclonal antibodies. M: Marker; lane 1: the expression products of BL21 (DE3) cells with pGEX-4T-1-CiCFI plasmid without IPTG induction; lane 2: the expression products of BL21 (DE3) cells with pGEX-4T-1-CiCFI plasmid induced by IPTG; lane 3: the purified rCiCFI protein; lane 4 and 5: the verification of rabbit anti-CiCFI and -CiC3 polyclonal antibodies detected by western blot; lane 6 and 7: the expression products of BL21 (DE3) cells with pGEX-4T-1 plasmid without or with IPTG induction; lane 8: the purified rGST protein.

A putative signal peptide containing 18 amino acids was predicted at the N-terminus of the CiCFI protein by the SignalP-5.0 tool (https://services.healthtech.dtu.dk/service.php?SignalP-5.0; accessed on 20 February 2020), with the probability of 0.6534 (Figure 5A). The recombinant plasmid (pGEX-4T-1-CiCFI) encoding the mature peptide of ^19^Leu-^706^Gln, rCICFI, was transformed into *E. coli* BL21 (DE3) cells. After the IPTG induction, the whole cell lysate was analyzed by SDS-PAGE, and a distinct band with the molecular weight of 100 kDa was revealed, which was consistent with the predicted molecular mass of CiCFI fused to a GST-tag (Figure 5B). To examine the cleavage effect of rCiCFI on C3b in the grass carp serum, anti-CiCFI and anti-CiC3 antibodies were prepared and their specificities were verified by western blot. The results showed that the sharp protein band of CiCFI was revealed in the grass carp serum by using anti-CiCFI antibodies; meanwhile, both the protein bands of CiC3 and CiC3b were also detected in the grass carp serum by using anti-CiC3 antibodies (Figure 5B). 

**Figure 6:**
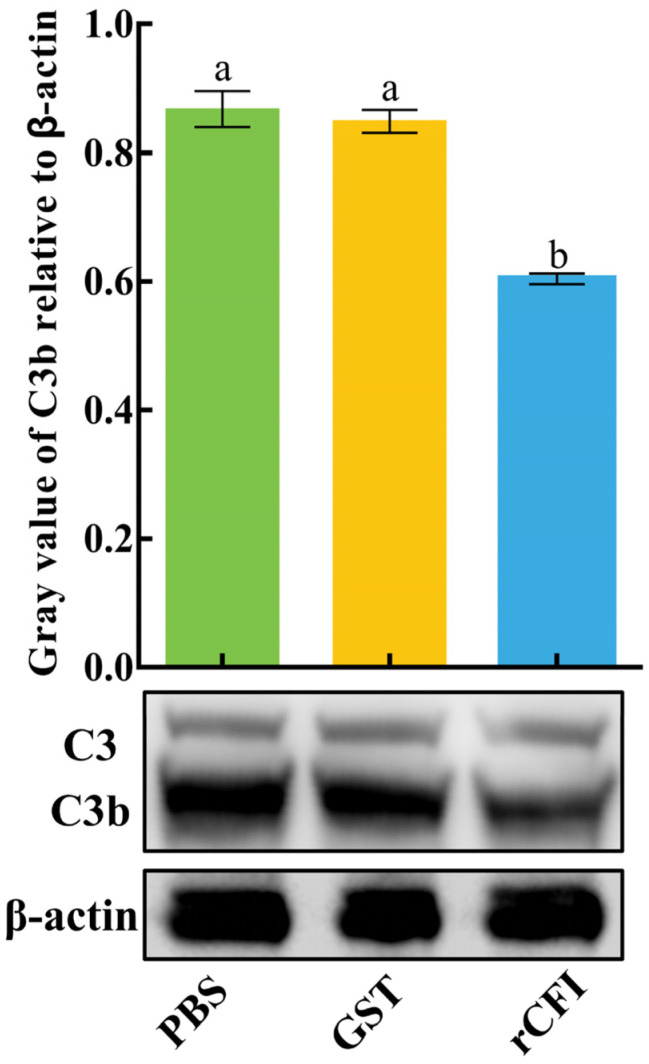
Degradative effect of rCiCFI on the CiC3b in the grass carp serum. Letters a and b indicate significant differences between various experimental groups (*p* < 0.05).

It has been reported that CFI can degrade C3b to prevent excessive activation of the complement system [12]. To verify whether the CiCFI protein exhibited this biological function, the purified rCiCFI protein was incubated with the grass carp serum at room temperature for 2 h, and the cleavage of CiC3b in the grass carp serum was detected by western blot. rGST proteins and PBS incubated with the grass carp serum were used as the negative and blank control group, respectively. The results showed that both the protein bands of C3 and C3b were obviously detected in the rCiCFI, rGST and PBS treated grass carp serum. Worthy of note, the relative content of C3b was significantly lower in rCiCFI treated serum than that in rGST treated serum (*p* < 0.05), whereas the relative content of C3b showed no significant difference between the rGST treated serum and the PBS treated serum (*p* > 0.05, Figure 6). 

### 2.5. The Distributions of CiCFI mRNA Transcripts and Proteins in Grass Carp Tissues

**Figure 7:**
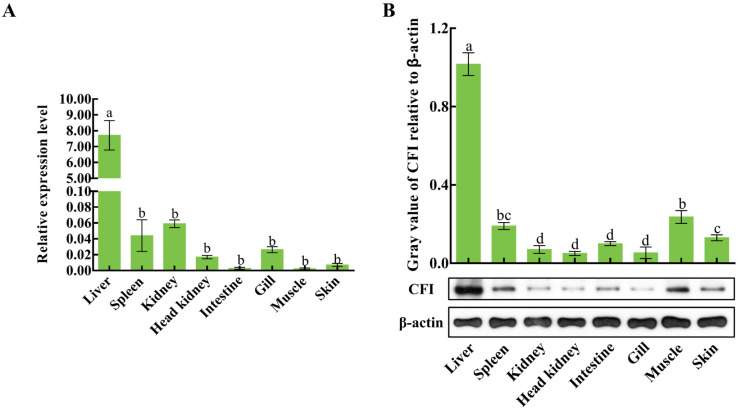
The mRNA and protein expressions of CiCFI in various tissues of grass carps. Letters a, b, c and d indicate significant differences among various tissues (*p* < 0.05). (**A**) The mRNA expressions of CiCFI in various tissues of grass carps. (**B**) The protein expressions of CiCFI in various tissues of grass carps.

The qPCR was carried out to detect the mRNA expression level of CiCFI in different tissues of uninfected grass carps, including the liver, spleen, kidney, head kidney, intestine, gill, muscle and skin. The results showed that the mRNA transcripts of CiCFI were widely detected in these eight tissues. In detail, the content of CiCFI mRNA transcripts was the most abundant in the liver, where its content was 3476.43-fold more than in the muscle (*p* < 0.05, Figure 7A). The CiCFI protein levels in the above eight tissues were also detected by western blot. In Figure 7B, the bands of CiCFI protein in the liver, spleen, kidney, head kidney, intestine, gill, muscle and skin were clearly revealed. After normalization with the band intensity of β-actin, it was presented that CiCFI protein showed the highest relative expression level in the liver (*p* < 0.05, Figure 7B), which was 20.33-fold of that in the head kidney with the lowest protein expression level (Figure 7B).

### 2.6. The mRNA and Protein Expression Changes of CiCFI in the Liver during GCRV Infection

**Figure 8:**
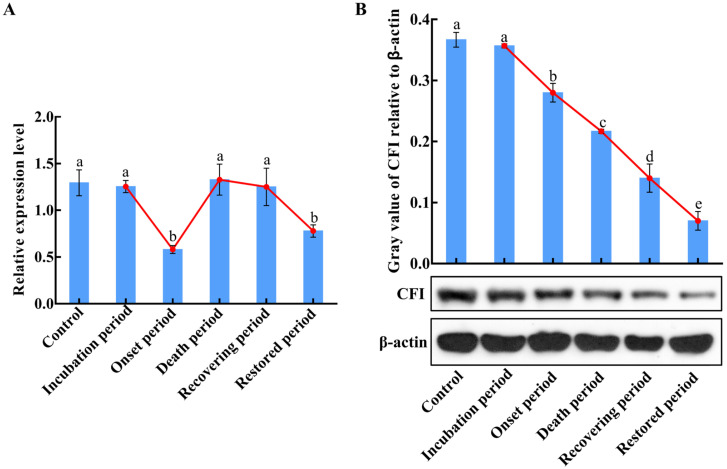
The mRNA and protein expression level changes of CiCFI in the liver of grass carps after GCRV infection. Letters a, b, c, d, and e indicate significant differences among the expressions in the liver at various periods post-GCRV infection (*p* < 0.05). (**A**) The mRNA expression level changes of CiCFI in the liver of grass carps after GCRV infection. (**B**) The protein expression level changes of CiCFI in the liver of grass carps after GCRV infection.

To investigate the dynamic synthetic characteristics of CiCFI during GCRV infection, the mRNA and protein expression levels of CiCFI in the liver of grass carps after GCRV infection at the incubation period, the onset period, the death period, the recovering period, and the restored period were analyzed by qPCR and western blot. The results displayed that the mRNA expression levels of CiCFI in the liver first decreased, then increased, and then decreased during GCRV infection (Figure 8A). Specifically, the mRNA expression level of CiCFI after GCRV infection at the incubation period showed no significant difference compared to the control (Figure 8A), while the mRNA expression levels of CiCFI significantly decreased to 0.45-fold of that in the control at the onset period, then recovered to the initial level at the death period and then decreased to 0.6-fold of that in the control at the restored period (Figure 8A). Interestingly, the relative expression levels of CiCFI protein in the liver showed an overall downward trend after GCRV infection, which was significantly decreased from the incubation period to the restored period (*p* < 0.05, Figure 8B).

### 2.7. The Fold Changes of CiCFI and CiC3b Proteins in the Serum during GCRV Infection

**Figure 9:**
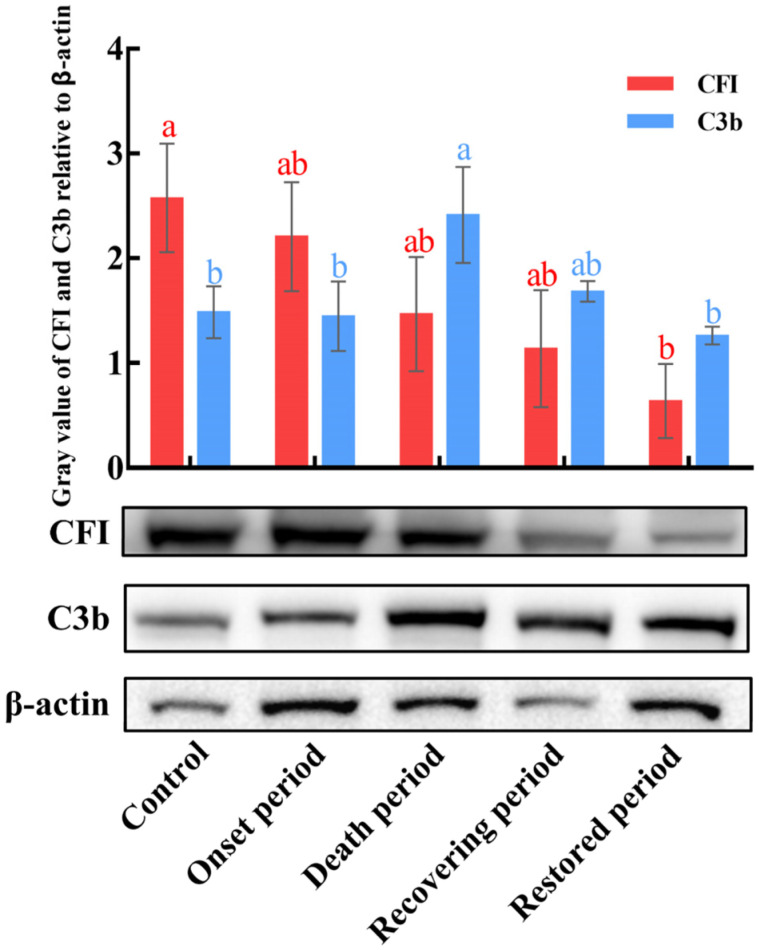
The protein content changes of CiCFI and CiC3b in the serum of grass carps after GCRV infection. Letters a and b indicate significant differences among the expressions in the serum at various periods post-GCRV infection (*p* < 0.05).

To explore the potential function characteristics and mechanisms of CiCFI during GCRV infection, relative protein levels of CiC3b and CiCFI in the serum were detected after GCRV infection at the incubation period, the onset period, the death period, the recovering period, and the restored period by western blot (Figure 9). Similar to the results in the liver during GCRV infection, the protein levels of CiCFI in the grass carp serum were significantly decreased from the incubation period to the restored period (*p* < 0.05, Figure 9), while the protein levels of CiC3b in the serum exhibited an upward trend and then a downward trend during GCRV infection (Figure 9). The protein level of CiC3b after GCRV infection at the incubation period showed no significant difference compared to the control (Figure 9). After GCRV infection at the death period, the protein level of CiC3b peaked, and it gradually recovered to the initial level at the restored period (Figure 9).

## 3. Discussion

Composed of more than 35 secreted plasma proteins, the complement system exerts efficient immune killing functions including the removal of aberrant cells and invading pathogens [36,37,38], the enhancement of inflammatory reactions [39,40], and modulation of innate as well as adaptive immune responses [41]. It has been accepted that there are three pathways of complement system activation marked by the cleavage of C3: the CP, the LP, and the AP [1]. The AP is regarded as the most ancient complement activation pathway from an evolutionary perspective, appearing early in echinoderms, and the most efficient complement activation pathway where the C3 is spontaneously cleaved [9,42,43]. In mammals, several complement-regulating proteins, including CFI, Df, complement factor P, etc., can strictly control the AP and balance the complement activation [13,44,45]. Teleost fish possess complement systems similar to those in mammals, and the identified fish complement proteins have many similarities to their mammalian counterparts [46]. However, few studies have been conducted on teleost fish complement-regulating proteins, particularly with regard to their functional aspects and regulatory mechanism during pathogen infection. In our previous study, transcriptome analyses show that the AP is significantly activated in grass carps after GCRV infection, with 2.18-fold up-regulation of Df mRNA expression and 9410.14-fold down-regulation of CFI mRNA expression [33]. Df, a complement enhancer, involves the initiation and amplification loops of complement system activation by cleaving Bf to promote C3 cleavage, and its regulation on complement activation has recently been verified in the grass carp [35]. CFI, a serine protease, is well-known as the key complement inhibitor, and plays a crucial role in host-pathogen interactions in mammals [13]. Nevertheless, the functional mechanisms of CFI in the complement system and in host-pathogen interactions remain largely unknown in teleost fish. In the present study, we cloned the full-length cDNA of CiCFI, ascertained its molecular and functional characteristics, investigated its mRNA and protein expression patterns in the liver and serum of grass carps after GCRV infection, and attempted to construct a full picture of the complement regulatory mechanism mediated by Df and CFI during GCRV infection by combining our previous results of Df in the grass carp. 

Conserved structures of CFIs have been revealed in vertebrates from cartilaginous fish to mammals [13,46,47]. These CFI homologs generally consist of five kinds of functional domains including the FIMAC domain, SR domain, LDLa1 domain, LDLa2 domain, and Tryp_SPc domain [13]. The Tryp_SPc domain is the functional core for CFIs as a serine protease to exert hydrolytic activity, and it is located in the light chain of CFIs, with the catalytic and substrate binding sites [48,49]. The other four kinds of domains, including the FIMAC domain, SR domain, LDLa1 domain, and LDLa2 domain constituting the heavy chain of CFIs, have also proven to be indispensable for CFI activation based on domain mutant experiments [48,50,51]. Between the heavy chain and the light chain of CFIs, there is a conserved cleavage site of Arg-Arg-Lys-Arg (RRKR). Upon the cleavage of this site, CFI protein is transformed from an inactive precursor into an active conformation [12]. In the present study, we found that the domain architecture of CiCFI was highly conserved, with a light chain, a heavy chain, and a cleavage site of RRKR. The light chain of CiCFI contained the Tryp_SPc domain with the catalytic triad (^504^His, ^551^Asp, ^648^Ser) and three residues of substrate binding site (^642^Asp, ^668^Ser, ^670^Gly), similarly to the CFIs reported from other vertebrates. Multiple sequence alignments revealed that 10 Cys residues of the FIMAC domain in the heavy chain were also conserved in CiCFIs. It has been shown that a subdomain within the FIMAC domain resembles the structure as a protease inhibitor of the follistatin, which hampers the activation of CFIs until the binding to a protein substrate complex induces conformational change in CFIs that allows proteolytic activity toward C3b [12,52]. These 10 Cys residues were considered the key to disulfide bridge formation and functional conformation of CFIs [15,53]. In addition, the amino acid sequence similarity comparison of CFIs showed that CiCFI shared high amino acid sequence similarity (50.4–78.7%) with other reported CFIs, although phylogenetic analysis indicated that CiCFI was evolutionarily close to the CFIs from teleost fish. These results collectively suggest the structural conservation of CiCFI, and its biological functions may also be conserved. Therefore, the research on the immune functions of CiCFI could refer to related studies on mammals or other vertebrates.

Upon the activation of the complement system, the human C3-α chain can be cleaved into C3b (177 kDa) by the C3 convertase; the C3b can be further fragmented to iC3b (175 kDa), C3c (135 kDa), C3dg (40 kDa), and then C3d (35 kDa) by CFI in the presence of certain cofactors, such as factor H [54,55,56]. In fact, C3b, the hydrolytic fragment of C3, serves as an opsonin in immunocytes phagocytosis and a fundamental element for C3 and C5 convertases in the AP of the complement system [57]. The fragments of C3b including C3c and C3d cannot be involved in the further complement cascade reaction, and the cleavage of C3b by CFI means the abatement of complement-mediated immune killing [54,58]. Given the crucial role in balancing complement activation, CFI is considered a vital target for pharmaceutical control in many complement-mediated autoimmune diseases in mammals [2,13]. In teleost fish, structural information and the mRNA expression profiles during pathogen infection are revealed in *I*. *punctatus* [22], *P*. *vachellii* [23], *O*. *mykiss* [25] and *C*. *carpio* [27]. In *C*. *semilaevis*, the CFI protein showed broad-spectrum antimicrobial activities against Gram-positive bacteria and Gram-negative bacteria [24]. The CFI in *P*. *olivaceus* also exhibited apparent binding capacities to a broad spectrum of bacteria and inhibited bacterial growth [26]. Recently, the interaction of CFI with C3 and the regulation on the deposition of C3 on the host cell surface have been verified in Cyclostomata *Lampetra morii* [47], but little research is focused on how CFIs regulate the complement activation in the more evolved teleost fish. It has been found only in *P*. *olivaceus* that the significant decrease in the serum C3b content is observed after the incubation of recombinant CFI proteins with the serum, thus confirming in *P*. *olivaceus* that CFI can negatively regulate complement activation by cleaving C3b [26]. The structure of C3 homologs seems to be diverse, with different amino acid sequence lengths, resulting in various sizes of C3’s stepwise cleavage products, including C3b, C3c, and C3d in different teleost fish [26,59]. Given the diversity of C3 and its stepwise cleavage products in teleost fish, detecting the content of serum C3b was adopted in the present study to examine the function of CFI according to the previous study in *P*. *olivaceus* [26]. The in vitro experiments indicated that the treatment of rCiCFI could significantly reduce the C3b protein content in the grass carp serum, similar with the CFIs in *P*. *olivaceus*, Cyclostomata and mammals. Collectively, the evidence demonstrates a conserved activity of CFI in degrading serum C3b in the grass carp.

The liver is an important immune tissue for teleost fish, synthesizing various immune-related proteins [60]. Previous studies have reported that CFI is mainly synthesized by hepatocytes in the liver in most vertebrates [61,62,63]. In the present study, we found that both the mRNA and protein expressions of CiCFI were highest in the liver of grass carp under normal physiological conditions, consistent with the results in previous studies. Therefore, CiCFI should also be mainly synthesized in the liver of grass carp. Currently, there are few studies on complement-reovirus interactions in mammals. Throughout research progress, however, it is shown that the relationship of complement-virus interactions is complex in mammals. Different mammalian virus infections involve different complement components. It has been recently demonstrated in humans that high levels of complement C3 were associated with persistent lung abnormalities in COVID-19 recovered subjects [64]. Fresh guinea pig serum can enhance the activity of immune serum against *Rous sarcoma* virus and this enhanced activity is tightly related to complements [65]. Remarkably, CFI, a complement inhibitor, has been clearly revealed to be sometimes hijacked by viruses and sometimes defend against viruses in mammalian hosts [18,20,66]. Therefore, CFI can be regarded as an important target to control virus infection in human medical research [18,21]. In teleost fish, the expression dynamics of CFIs have been investigated in immune tissues of several species during pathogenic infections, which may reflect the host complement system-pathogen interaction patterns. In *P*. *olivaceus*, for example, CFI mRNA expression showed a downward trend from 6 to 48 h in the spleen and kidney during *E*. *tarda* infection [26]. In *C*. *semilaevis*, CFI mRNA expression was also significantly down-regulated in the liver and intestine post *V*. *anguillarum* infection at 6 h, representing an early stage of infection, while sharply up-regulated at the late stage of infection [24]. The evidence reveals that the expression of CFI mRNA is down-regulated in the early stages of pathogen infection or disease onset and displays diverse expression patterns in response to various stimuli in teleost fish. These above mRNA expression changes of CFIs during bacterial infection were different from that in the present study. We detected the mRNA and protein expression levels of CiCFI in the liver of grass carp at five disease periods of GCHD previously described [67], including the incubation period, onset period, death period, recovering period, and restored period, to clarify the synthetic characteristics of CiCFI during GCRV infection. The results showed that both the mRNA and protein expression levels of CiCFI in the liver of grass carp significantly decreased in the onset period during GCRV infection, suggesting the distinct synthetic characteristics of CiCFI in the early stage of GCHD. Nevertheless, the mRNA and protein expression patterns of CiCFI differed at the late stage of infection where the mRNA expression returned to its initial level, while the protein expression level was down-regulated. The reason for this discrepancy may be that CiCFI proteins would be quickly secreted into the functional places including the serum where CFI proteins are exhausted to regulate complement activation during GCRV infection upon synthesis in the liver, since similar results have been reported [68,69]. Meanwhile, the results about the exhaustion of CiCFI proteins in the serum of grass carps during GCRV infection are also observed in our follow-up detection, probably supporting this explanation.

The serum of vertebrates contains more than 35 complement proteins, and is regarded as the functional place for the host complement system to eliminate the invading pathogens [1,9]. The form and content of complement components in the serum not only reflect the activation of the host’s complement system, but also represent the result of the host-pathogen interaction [70,71,72]. As the central component of the complement system, C3 is cleaved into C3a and C3b upon microbial pathogen invasion, and is often considered the marker of complement system activation [73,74]. To explore the potential functional characteristics and mechanisms of CiCFI in the complement system of grass carp during GCRV infection, the content of CiC3b and CiCFI in the serum were detected in the present study. The results showed that the content of CiCFI proteins continued to decline during GCRV infection from the early stage at the incubation period to the late stage at the restored period, corresponding to the results from the liver. In contrast, our previous study has revealed that Df, a well-known complement enhancer in the AP by cleaving Bf to promote the cleavage of C3 and the production of C3b, is observed with a continued up-regulated expression in the grass carp during GCRV infection, even at the late stage of infection [35]. Theoretically, the down-regulation of CFI and the up-regulation of Df should bring about an increase in the content of C3b proteins in the serum of grass carp during GCRV infection. Expectedly, the content of serum CiC3b proteins was first up-regulated and peaked at the death period in this study. However, the results also revealed that the content of CiC3b proteins returned to the initial level, although the content of CiCFI proteins was relatively low in the serum at the restored period. We speculate that there may be other complement inhibitors that also mediate the degradation and exhaustion of C3b in the serum of grass carp during GCRV infection in addition to CFI, so that the content of C3b can return to the initial level when the content of CFI is continuously down-regulated and the content of Df is continuously up-regulated. In mammals, CFI activity is regulated by cofactors (such as complement factor H, C4bp, CR1, etc.), so the cleavage of C3b is the result of the combined effects of CFI and cofactors [12,13]. Given that the fish genomes are equipped with the majority of human complement homologs including cofactors [5], we think CiCFI may also cooperate with certain cofactors to regulate the C3b content in the grass carp serum. Although the timing of the return of CiCFI levels to the initial level remains to be confirmed, the serum content analysis results collectively suggest the unique response characteristics of CiCFI in the complement system after GCRV infection and the complex regulatory mechanism of the complement system in the grass carp.

**Figure 10:**
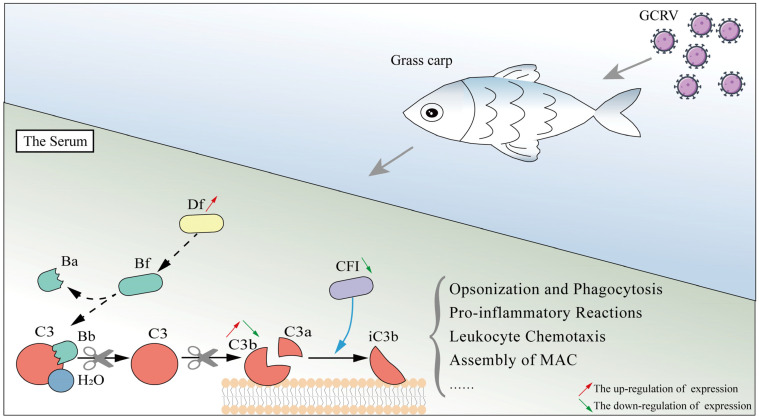
The schematic diagram of the complement regulatory mechanism mediated by Df and CFI during GCRV infection in *C. idella*. Df, a complement enhancer, shows continuously up-regulated expression levels in the grass carp during GCRV infection, and has previously been verified to promote the production of C3b, possibly by cleave Bf. In contrast, CFI, a complement inhibitor, continues to decline during GCRV infection from the early stage at the incubation period to the late stage at the restored period in the grass carp. Correspondingly, the content of serum C3b first increases and peaks at the death period, and eventually returns to the initial level at the restored period post GCRV infection in the grass carp. C3a and C3b produced by the cleavage of C3 can trigger diverse immune responses including opsonization and phagocytosis, pro-inflammatory reactions, leukocyte chemotaxis, the assembly of MAC, etc., in the grass carp serum.

In conclusion, this study identifies a complement inhibitor, CiCFI, from the grass carp *C. idella*. CiCFI is a serine protease characterized by a conserved functional Tryp_SPc domain, which harbors three residues of the catalytic triad and three residues of the substrate binding site. CiCFI proteins can promote the degradation of serum CiC3b, similar to what has been reported for other CFIs. The mRNA and protein expressions of CiCFI are the highest in the liver, which represents the main synthetic place of CiCFI in the grass carp. Unexpectedly, the protein levels of CiCFI were continuously down-regulated in the liver and serum of grass carp during GCRV infection, even at the late stage of infection, while the content of serum C3b proteins first increases and then returns to the initial level, suggesting a distinct role of CiCFI in regulating complement activation and fish-virus interaction. Combining the previously reported results that CiDf, a complement enhancer, shows continuously up-regulated expression levels, a schematic diagram about the functional and expressional patterns of Df and CFI in the grass carp during GCRV infection can be constructed (Figure 10). This study may provide an essential basis for the full picture of the complex complement regulation mechanism mediated by Df and CFI of the grass carp during pathogen infection.

## 4. Materials and Methods

### 4.1. Sequence Analysis of CiCFI

The full-length cDNA (GenBank: HM776035.1) and amino acid (GenBank: ADN22949.1) sequences of CiCFI were obtained from NCBI (https://www.ncbi.nlm.nih.gov/; accessed on 30 November 2019). The open reading frames (ORF) and deduced amino acid sequence of CiCFI were analyzed with Expert Protein Analysis System (http://www.expasy.org/; accessed on 30 November 2019). The isoelectric point and the molecular weight of deduced amino acid sequences were predicted at the ExPASy site (http://web.expasy.org/compute_pi/; accessed on 30 November 2019). The signal peptide was predicted by using the SignalP-5.0 tool (https://services.healthtech.dtu.dk/service.php?SignalP-5.0; accessed on 20 February 2020). Multiple sequence alignment and comparative homology analysis of CFIs from different species including *C. idella* (ADN22949.1), *D. rerio* (XP_017209949.1), *C. gibelio* (AGU16535.1), *C. Carpio CFI-A* (BAB88920.1), *C. Carpio CFI-B* (BAB88921.1), *A. testudineus* (XP_026225021.1), *Oryzias latipes* (XP_004079594.1), *C. semilaevis* (AKN79751.1), *Ginglymostoma cirratum* (ABV21980.1), *X. laevis* (NP_001079421.1), *G. gallus* (NP_001258947.1), *M. musculus* (NP_031712.2), *Bos taurus* (NP_001033185.1), and *H. sapiens* (NP_000195.2) were performed by Clustal Omega (https://www.ebi.ac.uk/Toolsmsa/clustalo/; accessed on 12 November 2021) and MatGat program Version 2.03 (Montclair State University, Montclair, NJ, USA), respectively. Clustal alignment results were presented and annotated by using Jalview Version 2.11 (The Barton Group, University of Dundee, Dundee, UK).

### 4.2. Structural and Phylogenetic Analysis of CiCFI

The domain architectures were predicted by the Simple Modular Architecture Research Tool (http://smart.embl-heidelberg.de/; accessed on 12 November 2021). The three-dimensional structure model was predicted by I-TASSER (http://zhanglab.ccmb.med.umich.edu/I-TASSER/; accessed on 12 November 2021) protein modeling server with the human CFI (GenBank: NP_000195.2) as the template and visualized with PyMOL software Version 2.4.0 (DeLano Scientific LLC, San Carlos, CA, USA). 

A phylogenetic tree was constructed by using MEGA software Version 7.0 (Pennsylvania State University, PA, USA) based on full-length amino acid sequences of CFI from different species (information shown in Table 1), and 1000 bootstrap replicates were set to assess the reliability of the branching. The phylogenetic tree was displayed and annotated by using iTOL (https://itol.embl.de/upload.cgi; accessed on 12 November 2021).

### 4.3. Prokaryotic Expression and Purification of Recombinant CiCFI Protein

The cDNA fragment encoding the mature peptide of CiCFI (^19^Leu–^706^Gln) was amplified by using 2 × Taq Plus Master Mix II (Vazyme, Nanjing, China) with specific primers of CiCFI-IF-F and CiCFI-IF-R (Table 2). The PCR fragment was digested by using the *BamH* I and *EcoR* I restriction enzymes (Takara, Kyoto, Japan) and ligated into the expression vector pGEX-4T-1 (Vazyme, Nanjing, China). The recombinant plasmid of pGEX-4T-1-CiCFI was transformed into *Escherichia coli* BL21 (DE3) cells (Novagen, Darmstadt, Germany), and the transformants were validated by sequencing. Positive transformants were incubated in LB medium (containing 100 μg/mL ampicillin) at 37 °C with shaking at 180 rpm. When the OD_600_ reached 0.5–0.6, 0.5 mM isopropyl-β-D-1-thiogalactosidase (IPTG) was added. After inducible expression for 24 h, the bacterial culture was sonicated and centrifuged to obtain the supernatant. The supernatant was further filtered by using a 0.45 µm filter membrane for protein purification. rCiCFI was purified by using a GST resin column and dialyzed against PBS buffer at 4 °C for 24 h. The induced bacteria lysate and purified recombinant protein were examined by SDS-PAGE (GenScript, Nanjing, China) and visualized with Coomassie Brilliant Blue R-250 (Bio-Rad, Hercules, CA, USA), and the concentration of the recombinant protein was measured by using the Pierce BCA Protein Assay Kit (Thermo Fisher Scientific, Waltham, MA, USA). The obtained protein was stored at −20 °C for subsequent experiments.

### 4.4. Preparation and Validation of Polyclonal Antibodies

The cDNA sequence encoding the protein fragment of CiCFI (^400^Gly–^560^Leu) was cloned and recombined to obtain the antigen proteins through prokaryotic expression as in the above descriptions. This purified recombinant protein fragment (1 μg/μL) was used to immunize Japanese white rabbits and prepare polyclonal antibodies referring to the previous report [75]. After four immunizations with antigen proteins and adjuvant (Sigma-Aldrich, St. Louis, MO, USA), the serum of rabbit was collected and then purified as in the rabbit anti-CiCFI polyclonal antibodies according to the protocol from IgG Purification Kit-G (Dojindo, Kumamoto, Japan). For the preparation of rabbit anti-grass carp C3 (designated as CiC3) polyclonal antibodies, Keyhole limpet hemocyanin (KLH)-conjugated peptide of CiC3 (GenBank: AAQ74974.1, ^1350^Glu–^1365^Asp) was synthesized and employed as the antigen proteins.

Western blot analysis was conducted to determine the specificity of the polyclonal antibodies against CiCFI and CiC3. Proteins from the blood of healthy grass carp individuals were isolated by using an ice-cold RIPA lysis buffer containing phenylmethanesulfonyl fluoride (Beyotime, Shanghai, China). The protein bands were separated by 10% SDS-PAGE (Beyotime, Shanghai, China) with DTT in an SDS-PAGE sample loading buffer (Beyotime, Shanghai, China) and transferred to a polyvinylidene difluoride membrane (Millipore, MA, USA). The membranes were blocked in QuickBlock Blocking Buffer (Beyotime, Shanghai, China) for 15 min at 25 °C and incubated with the rabbit anti-CiCFI polyclonal antibody (diluted 1:1000 in QuickBlock Blocking Buffer) or rabbit anti-CiC3 polyclonal antibody (diluted 1:1000 in QuickBlock Blocking Buffer) overnight at 4 °C, respectively. After being washed three times with 1 × Tris-buffered saline containing 0.05% Tween-20, the membrane was incubated with the 1:2000 diluted HRP-conjugated goat anti-rabbit IgG (Abclonal, Wuhan, China) at 25 °C for 1 h. Finally, the membranes were incubated in the BeyoECL Plus substrate system (Beyotime, Shanghai, China) for imaging under the GeneSys Imaging System (Alcatel, Paris, France). 

### 4.5. The Incubation of rCiCFI with Grass Carp Serum

The activity of rCiCFI to degrade serum C3b protein was analyzed according to previous descriptions [12]. The grass carp serum was diluted with 20-fold DGVB buffer (containing 2.5 mM sodium barbital, 71 mM NaCl, 0.15 mM CaCl_2_, 0.5 mM MgCl_2_, 2.5% *w*/*v* glucose, 0.1% *w*/*v* gelatin, pH 7.4) and mixed with rCiCFI or GST (negative control) to the final concentration of 40 μg/mL, followed by the incubation at room temperature for 2 h. The experimental procedures were conducted according to the previous descriptions [76]. In this experiment, we did not detect intrinsic CFI, but we could ensure that all serum samples were detected at the same dilution. The protein content of CiC3b in the serum was then detected by western blot as described above. The band intensity was quantified and analyzed by using Image J software Version 1.48 (NIH, Bethesda, MD, USA). The β-actin was used as an internal reference protein. 

### 4.6. The Expression Analysis of CiCFI mRNA and Protein Expression in Different Tissues

For the expression analysis of CiCFI mRNA and protein expression in different tissues, the liver, spleen, kidney, head kidney, intestine, gill, muscle and skin were sampled from three uninfected grass carp individuals. 

Tissue total RNA was extracted by using an E.Z.N.A.^®^ Total RNA Kit Ⅱ (Omega, Norcross, GA, USA). RNA was treated with DNase I, and cDNA was synthesized by using a RevertAid™ First Strand cDNA Synthesis Kit (Thermo Fisher Scientific, Waltham, MA, USA), according to the manufacturer’s instructions. qPCR was conducted to investigate mRNA expression levels of CiCFI in various tissues. The qPCR was performed on the CFX96 Touch^TM^ Real-Time PCR Detection System (Bio-Rad, Hercules, CA, USA), with a total volume of 10 μL containing 5 μL ChamQ^TM^ Universal SYBR^®^ qPCR Master Mix (Vazyme, Nanjing, China), 1 μL of diluted cDNA template, 0.8 μL primer mixture, and 3.2 μL ddH_2_O. The qPCR programs were set as follows: 95 °C for 10 min, followed by 35 cycles at 95 °C for 10 s, 60 °C for 10 s, and 72 °C for 15 s, and followed by a dissociation curve analysis (raised from 65 °C to 95 °C with an increase of 0.5 °C every 5 s) to verify the amplification of a single product. The relative expression level of the target gene was normalized against the expression level of the *β-actin* and the *18S rRNA*. The data were analyzed with the 2^–ΔΔCt^ method [77]. All primers for qPCR were listed in Table 2. 

Tissue proteins were isolated by using an ice-cold RIPA lysis buffer containing phenylmethanesulfonyl fluoride. Western blot analysis was executed to detect the protein expression level of CiCFI in different tissues. The β-actin was used as an internal reference protein.

### 4.7. The Detection of CiCFI mRNA and Protein Level in the Liver after GCRV Infection

Grass carps with an average body weight of 60 ± 6.4 g were collected from a fish farm in Changsha City, Hunan Province, China. The fish were temporarily reared in a tank for two weeks at a constant temperature of 28 °C and fed twice a day at 2% of their body weight prior to use in experiments. A total of 60 grass carp were employed for the GCRV challenge experiment, and they were randomly divided into two groups. One group was intraperitoneally injected with 10^7^ TCID_50_/mL of GCRV-AH528 previously identified in our lab and set as the experimental group. The other group was intraperitoneally injected with the equivalent volume of PBS buffer for use as the control group. After GCRV challenge, samples from five individuals were sampled at five periods according to the previous study [68], including the incubation period (12 h post GCRV challenge, before the occurrence of GCHD’s symptoms), onset period (GCHD symptoms were emerging), death period (grass carps began to die), recovering period (grass carps began to recover), and restored period (grass carp recovered completely and the symptoms of GCHD were disappeared). Because the liver and serum were reported as the main sites where CFI was synthesized and exerted biological functions, respectively, the tissues including the liver and blood were sampled from the grass carps in the experimental group and control group. To reveal the synthetic characteristics of CiCFI, the mRNA and protein expression changes of CiCFI in the liver during GCRV infection were analyzed by qPCR and western blot as in the above descriptions. 

### 4.8. The Detection of CiCFI and CiC3b Protein Levels in the Serum after GCRV Infection

The blood samples from the experimental group and control group grass carps were stored at 4 °C overnight for blood coagulation, followed by the centrifugation at 5000× *g* to obtain the upper serum. To investigate the potential function characteristics of CiCFI during GCRV infection, CiCFI and CiC3b protein levels in the serum were detected by western blot at four infection periods including onset period, death period, recovering period, and restored period. The β-actin was used as an internal reference protein.

### 4.9. Statistical Analysis

All data were represented as mean ± standard error (*N* = 3 or 5). The differences in relative mRNA and protein levels in the experimental and control groups were compared by one-way analysis with variance and Duncan’s post-hoc test. All of the statistical analyses were conducted by using Statistical Package for Social Sciences Version 25.0 (SPSS Inc., Chicago, IL, USA). A *p*-value < 0.05 was considered statistically significant.

## Figures and Tables

**Table 1:** The information of CFIs from different vertebrates used in phylogenetic analysis.

Species	Accession Number
*Lethenteron camtschaticum*	BAG66070.1
*Homo sapiens*	NP_000195.2
*Pongo abelii*	XP_024101436.1
*Bos taurus*	NP_001033185.1
*Mus musculus*	NP_031712.2
*Rattus norvegicus*	NP_077071.1
*Gallus gallus*	NP_001258947.1
*Xenopus laevis*	NP_001079421.1
*Chiloscyllium plagiosum*	AHA61785.1
*Ginglymostoma cirratum*	ABV21980.1
*Oncorhynchus mykiss*	XP_021449058.1
*Esox lucius*	XP_028973634.1
*Oryzias latipes*	XP_004079594.1
*Cynoglossus semilaevis*	AKN79751.1
*Echeneis naucrate*	XP_029381915.1
*Monopterus albus*	XP_020477827.1
*Oreochromis niloticus*	XP_005459189.1
*Anabas testudineus*	XP_026225021.1
*Perca flavescens*	XP_028420661.1
*Ictalurus punctatus*	ACV87004.1
*Tachysurus vachellii*	ATC38299.1
*Tachysurus fulvidraco*	XP_027018314.1
*Electrophorus electricus*	XP_026852291.1
*Cyprinus carpio*	BAB88920.1/BAB88921.1
*Carassius gibelio*	AGU16535.1
*Labeo rohita*	RXN08433.1
*Danio rerio*	XP_017209949.1
*Anabarilius grahami*	ROL01506.1

**Table 2:** Description of primers used in this study.

Primer Name	Primer Sequence 5′-3′	Usage	Accession
CFI-IF-F	GATCTGGTTCCGCGTGGATCCCTGAAGGAACTATCAGAGCT	CDS amplification	HM776035.1
CFI-IF-R	CTCGAGTCGACCCGGGAATTCCTGGTTATATTTGGTTACAG	CDS amplification	
CFI-F	CACATAACGTACTATTGGCAAC	qPCR	
CFI-R	CCGACATTGAGTGATGACCA	qPCR	
β-actin-F	GCTATGTGGCTCTTGACTTCG	qPCR	M25013.1
β-actin-R	GGGCACCTGAACCTCTCATT	qPCR	
18S rRNA-F	ATTTCCGACACGGAGAGG	qPCR	EU047719.1
18S rRNA-R	CATGGGTTTAGGATACGCTC	qPCR	

Note: F—forward primer; R—reverse primer.

## Data Availability

The data presented in this study are available on request from the corresponding author.
